# The biopsychosocial benefits of amputee soccer: perspectives from members of the American Amputee Soccer Association

**DOI:** 10.3389/fspor.2026.1791418

**Published:** 2026-03-25

**Authors:** Daniel Joseph Lee, Rosalia Casanova, James Pierre-Glaude, Eric M Lamberg

**Affiliations:** Stony Brook University, Stony Brook, NY, United States

**Keywords:** advocacy, amputee soccer, biopsychosocial, community, limb loss, adaptive sports

## Abstract

**Background:**

Individuals with limb deficiency and limb loss (LD/LL) may suffer a negative impact to their biopsychosocial function as a result of their disability. Adaptive sports is a recognized avenue in which individuals with disability can improve their biopsychosocial function beyond what is experienced without participation. Our study explored the impact of participating in amputee soccer on biopsychosocial function from the perspective of the playing members of the American Amputee Soccer Association (AASA).

**Methods:**

The study utilized a qualitative design and followed the Standards for Reporting Qualitative Research. Data were collected using survey questions and semi-structured interviews. Data were analyzed using descriptive statistics for survey data and inductive coding to generate codes and themes.

**Results:**

Fifteen participants completed both the survey and interview. Survey results were consistent with a high quality of life. Three themes were generated from the data: 1) More than a Sport, it is a Community, 2) Benefits Body and Mind, and 3) Call to Action: Advocating for Others.

**Conclusion:**

Participants in the AASA reported a high quality of life and positive biopsychosocial benefits from playing amputee soccer. The benefits of participating in amputee soccer may exceed participating in a support group alone.

## Introduction

Adaptive sports provide the opportunity for people living with disability to participate in a variety of physical activities. From recreational activities to elite competition, participation in adaptive sports benefits their physical health, quality of life, and sense of community (1–3). Adaptive sports for individuals with disabilities were first introduced in the mid-20th century as a rehabilitation mechanism for injured war veterans. Today, adaptive sports have progressed to include all ages, abilities, and nearly all sports; from recreational to national, international and Paralympic competitions.

Individuals living with limb difference or limb loss (LD/LL) are one subsection of the population that engages in adaptive sport (4). In the United States there are 5.6 million people living with LD/LA. On average, 42,650 born with limb difference and 464,644 with acquired limb loss annually (5). One of the fastest growing adaptive sport opportunities for those living with LD/LA is amputee soccer. Amputee soccer is a unique form of adaptive sports in that it allows both those with upper and lower LD/LL participate on relatively equal terms. Players with upper LD/LL participate as goalkeepers and are only allowed to use their sound upper limb to save the ball. Players with LD/LL participate as field players using their sound limb to control/strike the ball and bilateral forearm crutches to move about the pitch. Players are not permitted to use prosthetic devices on the field, which negates the impact of technology on the performance. There are at least 47 participating nations recognized as part of the World Amputee Football Federation (WAFF). In the United States, the American Amputee Soccer Association (AASA) is the sole recognized member of WAFF.

Individuals living with LD/LL may face significant challenges to their biopsychosocial function. Biopsychosocial function encompasses all aspects of human function and belonging and is made up of the three intertwined domains of biological, psychological and social functioning (6). For those with LD/LL, the biological function is perhaps the most readily recognized given the absence of all or part of a limb. Less apparent can be the impact of living with LD/LL on the psychosocial domains. This impact may present in the form of depression, anxiety, social isolation or withdrawal, cognitive impairment, and a decreased quality of life (7–9). However, participating in an adaptive sport like amputee soccer may effectively mitigate some of these biopsychosocial deficiencies.

A study by Yazicioglu et al. in 2007 found that amputee soccer players had not only greater balance and strength than the control group (consisted of individuals with LD/LL that did not participate in amputee soccer), but also had significantly higher quality of life metrics (10). High quality of life scores were additionally found in a study by Auricchio et al. in 2017, showing that participants in amputee soccer had greater biopsychosocial function than non-participating counterparts (11). Furthermore, a study by Monteiro et al. found that participants in amputee soccer showed higher scores on all domains of biopsychosocial function when compared to non-participants of any sport (12).

Since it is known that individuals living with LD/LA can experience significant negative impacts to their biopsychosocial function, and that participating in amputee soccer can help improve biopsychosocial function of those with LD/LA, we wanted to explore how participation in the sport impacted the biopsychosocial function. Specifically, we wanted to look at members of the AASA as this group has yet to be studied. Therefore, the purpose of this study was to explore how participating in amputee soccer as a member of the AASA impacted biopsychosocial function.

## Methods

### Study design

The study utilized a qualitative design.

### Reporting

The Standards for Reporting Qualitative Research (SRQR) were used in the formatting of this manuscript (13).

### Participants

To be eligible to participate in the study, participants needed to:

- Be a member of the American Amputee Soccer Association
- Have at least 6 months of active participation
- Participated in at least 3 events or practices
- 18 years old or older
- Speak either English or Spanish

The rationale for the inclusion criteria was to ensure participants had enough experience with amputee soccer to be able to contribute to the themes developed in this study. A sample size of 15 participants was projected for the study this sample size was believed to be sufficient to achieve data saturation given the narrow scope of the subject matter and relative homogeneity of the participants. Data saturation is established when there is consistency of responses, and the sample size chosen for this study follows established sample sizes for qualitative research presented in the literature (14).

Participants were recruited for this study from the AASA via an email solicitation. Interested participants who were eligible to participate in the study were provided a direct link to the survey and video conferencing interview.

### Procedure

A survey and a semi-structured interview script were developed for use in this study through a collaborative effort of the research team and a literature review related to the biopsychosocial aspects of participating in adaptive sports. The survey consisted of a total of 50 questions and was estimated to take 20 min to complete using Qualtrics XM (Provo, UT). Prior to beginning the survey and following the consent form, participants had to attest to meeting the inclusion criteria outlined above. Thirteen items on the survey pertain to demographic variables (Supplementary Material A1), 11 Likert-like questions regarding opinions on amputee soccer (Supplementary Material B1), and the final part of the survey utilized the World Health Organization’s Quality of Life-Brief (WHOQOL-BREF) instrument (Supplementary Material C1), an abbreviated version of the World Health Organizations Quality of Life Assessment. (WHOQOL Group). The interview consisted of 13 questions focused on amputee soccer participation (Supplementary Material D1) and was estimated to take 30 min to complete using Zoom (San Jose, CA). Interviews were one on one and were audio recorded. Field notes were generated by the interviewer during each session and probing follow-up questions were used when ambiguity in a participant's response was present.

One member of the research team (RC) conducted all of the interviews following training from an experienced qualitative researcher. Once the interview script and survey were drafted, it was piloted with an individual who has participated in amputee soccer in the past and who was not participating in the study. The time to complete the entirety of the study was under one hour. No further revisions were made to the instruments following the pilot and the materials were then translated into Spanish. After the interviews were completed, the recordings were transcribed by a third party unrelated to the study. Transcripts were reviewed by the researchers to ensure accuracy prior to coding.

### Ethical review

Prior to the beginning of the study, the Institutional Review Board at Stony Brook University approved this study. Informed consent was given prior to conducting research.

### Data analysis

The transcribed interviews were analyzed independently by three members of the research team using an inductive coding approach. The inductive approach allows for unbiased interpretation of the transcripts without any preordained goal. This approach can decrease researcher bias and allow the results to better reflect the participant's voices (15). The coded transcripts were then individually discussed by all three coders until agreement was reached. Once the codes were agreed upon, the four-member research team thematically analyzed the codes to form themes using the consensus method. The data were validated by utilizing field notes and audio recordings, when needed.

## Results

**Figure 1:**
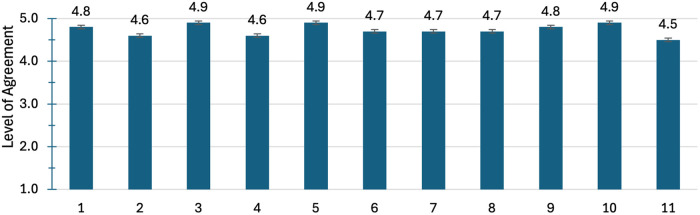
Survey questions from Supplementary Material B1. A higher score (5/5) indicates a higher level of agreement.

**Figure 2:**
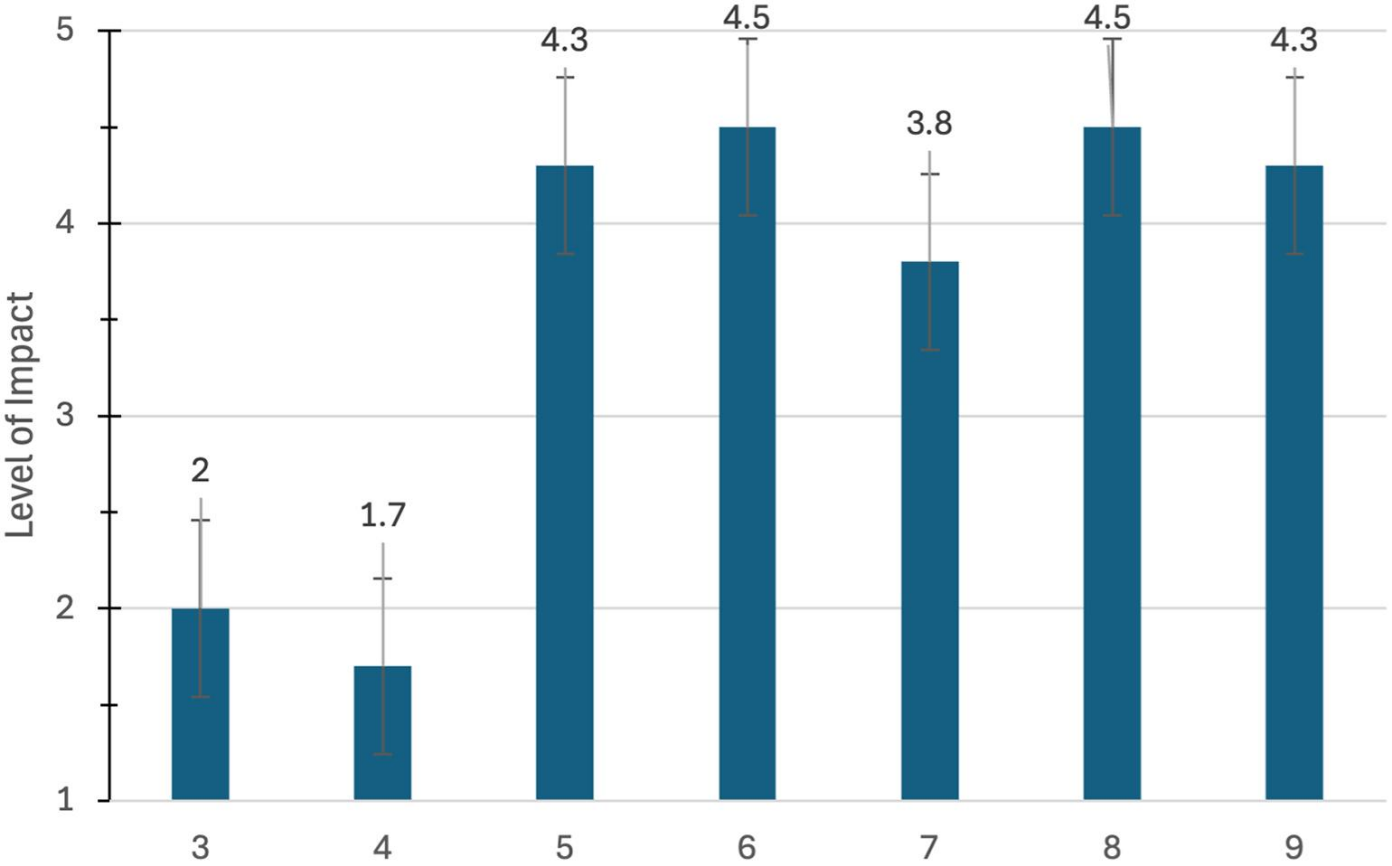
Questions from the WHOQOL-BREF with a higher score (5/5) being desirable for questions 5–9 and a lower score (1/5) being optimal for questions 3 and 4.

**Figure 3:**
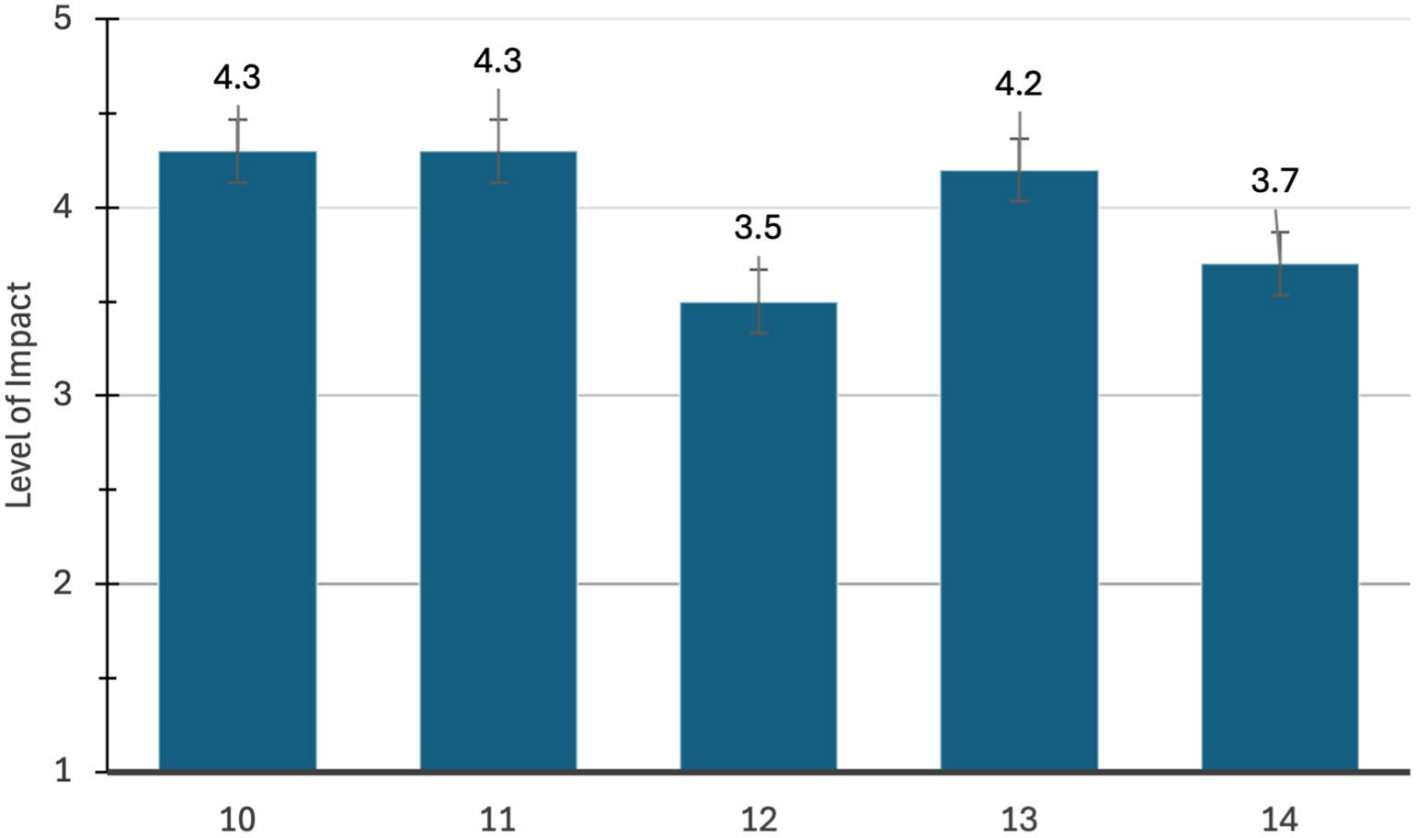
Questions from the WHOQOL-BREF with a higher score (5/5) being desirable.

**Figure 4:**
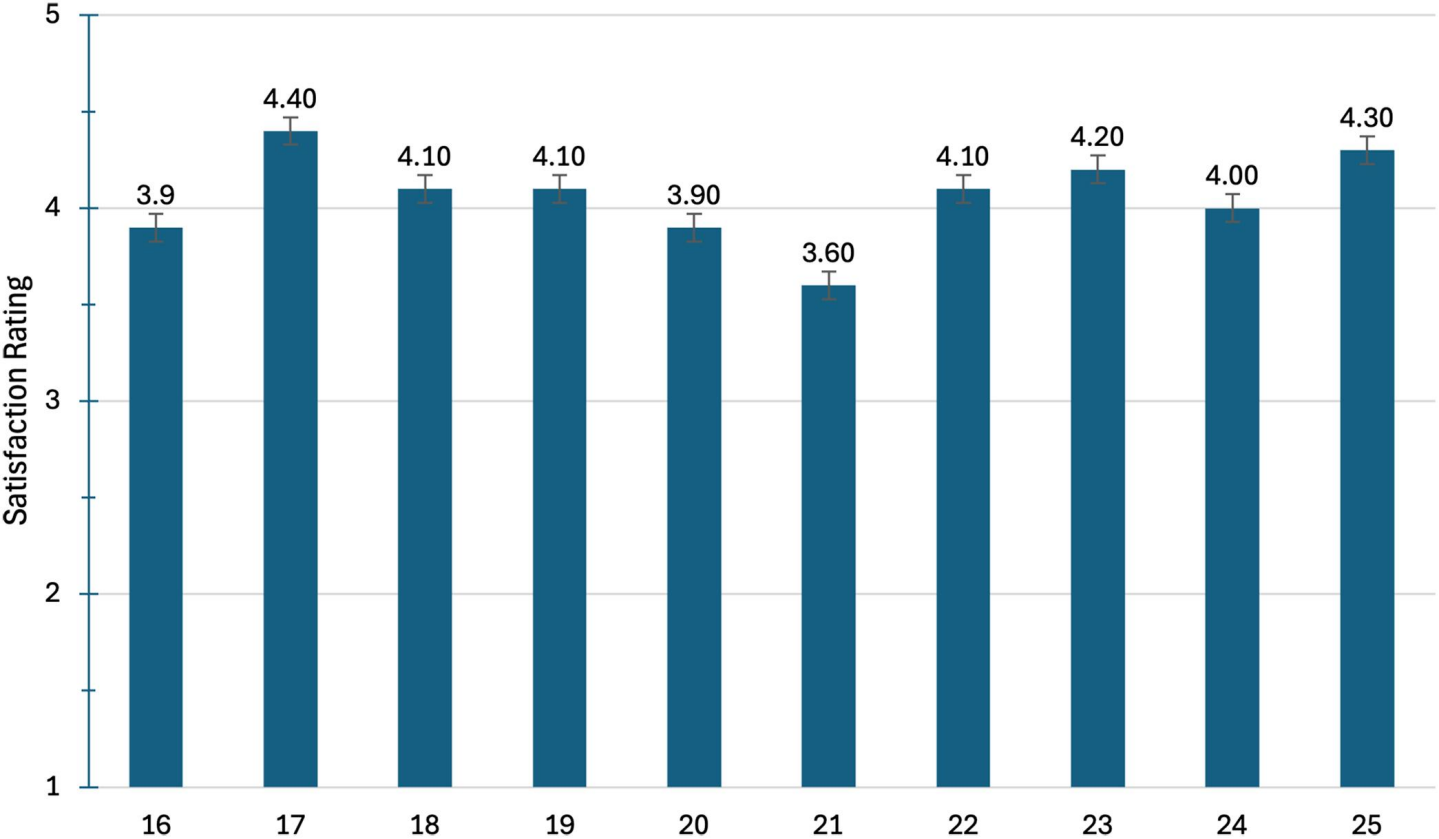
Questions from the WHOQOL-BREF with a higher score (5/5) being desirable.

The survey and interview were available for four months between April and August of 2024. Fifteen participants completed the survey and the interviews. The average time to complete the survey and the interviews were 9.5 and 33.3 min, respectively. Demographic variables are presented in Table 1. Mean scores on survey questions are presented individually in Figures 1–4.

**Table 1:** Demographic variables (*n* = 15).

Demographic variables	Frequency
Age [mean (SD) years]	36.94 (9.69)
Female [% (number)]	33.00% (5)
Employed full-time [% (number)]	73.33% (11)
Level of limb loss/difference (number)	TT: 6
	TF:2
	HD:3
	KD:3
	AD:1
Etiology of limb loss/difference (number)	Infection: 3
	Cancer: 5
	Trauma: 5
	Congenital: 2
Time since limb loss (years)	0–1 year: 1
	2–5 years: 5
	6–10 years: 2
	11–15 years: 1
	16–20 years: 2
	20+ years: 4
Hours of prosthesis use (daily average)	0–2 hrs/day: 4
	6–13 hrs/day: 5
	14+ hrs/day: 6
Length of playing amputee soccer (years)	0–1 years: 5
	2–5 years: 4
	6–10 years: 3
	15+ years: 3

TT, transtibial; TF, transfemoral; HD, hip disarticulation; KD, knee disarticulation; AD, ankle disarticulation.

Participants scored highly on the WHOQOL-BREF and survey questions. Figure 1 shows scoring of participants on the survey questions (Supplementary Material B1). The results were that participants scored between “agree” and “strongly agree” on all questions. This indicates that participating in amputee soccer has improved their: overall life satisfaction, engagement and interest in leisure activities, sense of community, support network, sense of belonging, self-confidence, perception of self, sense of purpose, overall physical fitness, physical activity level, and energy levels.

Figure 2 scales between “not at all” (1) and an “extreme amount” (5) on a subset of questions (questions 3–9) related to quality of life over the last two weeks on the WHOQOL-BREF (Supplementary Material C1). Participants scored primarily between “very much” and “an extreme amount” on four of the questions (#’5,6,8,9) One question (#7) scored below the 4.0 threshold of “very much” regarding the ability to concentrate. For questions 3 and 4, a lower score was desirable, indicating that participants were between “not at all” and “a little” regarding physical pain and medical treatments required to function, respectively.

Figure 3 represents the participant's responses on the WHOQOL-BREF related to ability to do things over the last two weeks, with a higher score being desirable. For questions 10,11, and 13, results were between “mostly” and “completely”. Questions 12 and 14 were scored between “moderate” and “mostly” regarding finances and leisure opportunities, respectively.

Figure 4 illustrates the average score value between “satisfied” and “very satisfied” for all questions except three on the WHOQOL-BREF. The questions that were below the 4.0 threshold for “satisfied” were regarding satisfaction of sleep (#16), in personal relationships (#20), and sex life (#21).

Certain survey questions used a different scale than the others, and the results for these are presented independent of the figures. All questions used a Likert-like scale with ratings ranging between 1-5, however, the scale was inverted or altered from those presented in the figures for the following items on the WHOQOL-BREF. Question 1 asked participants to rate their quality of life. Participants scored a mean (SD) value of 4.40(.74) indicating responses between “good” and very good”. Question 2 asked about health satisfaction and was scored at a mean of 4.20(.77) indicating scores between “satisfied” and “very satisfied”. Question 15 asked about a participant's mobility and had a mean value of 4.87(.35) corresponding closely to responses of “very good”. Finally, for Question 26, participants responded with an average of 2.40(1.12) about the presence of negative feelings over the past two weeks. This value lies between “seldom” and “quite often”.

### Qualitative results

From the coded transcripts, three themes were developed: 1) More than a Sport, it is a Community, 2) Benefits Body and Mind, and 3) Call to Action: Advocating for Others. Individual participant characteristics are shown below in Table 2.

**Table 2:** Participant characteristics.

Participant name	Age	Gender	Amputation level	Experience with AASA
“Ashley”	33	Female	Transtibial	<1 year
“Mike”	26	Male	Knee disarticulation	2–5 years
“Rose”	38	Female	Hip disarticulation	<1 year
“Kyle”	32	Male	Transfemoral	6–10 years
“Jack”	47	Male	Transtibial	2–5 years
“Max”	30	Male	Transtibial	6–10 years
“Sally”	44	Female	Hip disarticulation	<1 year
“Josh”	29	Male	Hip disarticulation	15+ years
“Laura”	37	Female	Knee disarticulation	2–5 years
“Sophia”	41	Female	Transtibial	<1 year
“Chris”	45	Male	Knee disarticulation	2–5 years
“Gary”	60	Male	Transtibial	15+ years
“Jose”	39	Male	Transtibial	15+ years
“Giovanni”	40	Male	Transfemoral	6–10 years
“Luis”	31	Male	Ankle disarticulation	<1 year

#### Theme 1: more than a sport, it is a community

On the surface, amputee soccer, like many other adaptive sports, is focused primarily on performance and competition. However, the participants in this study described a phenomenon that transcends the sport itself into the realm of community and belongingness. Participants remarked that amputee soccer created an anchor point for their lives. Many remarked that following their limb loss (or having grown up with limb difference) that they can feel isolated from their peers and society. This sense of isolation was not necessarily mitigated through traditional support groups, rather, it was not until they participated in amputee soccer that they found their “people”. This was described by participants as having a community of similar people, not just because of their physical presentation, but also because of their shared goals and values.

Sally (44 y.o. female, <1 year of experience with the AASA, hip disarticulation) encompasses this sentiment in the following statement:

“I don’t feel like I’m out on an iceberg floating by myself going, ‘Well, I’m not really sure how I’m gonna do this.’ Now, it feels more like there are people on the shore that I can say, ‘Hey, I’m drifting away, pull me in.’ And there are people who will understand exactly what I mean, and have some way of pulling that iceberg in, if that makes sense.”

Ashley (33 y.o. female, <1 year of experience with the AASA, transtibial) describes how disability becomes relative once you are part of amputee soccer:

“I feel free when I have the ability to run. It gives me a time where everybody is the same and nobody on the field has a disability, because everybody is the same. So, no one is the disabled person, because everybody is the disabled person.”

Chris (45 y.o. male, 2-5 years’ experience with the AASA, knee disarticulation) shares how the community acts as a way of transitioning from before trauma to afterwards:

“Finding a community of people to connect with has been very helpful. And I think the other thing too is having a traumatic event happen to you like I did, it’s very easy to sort of shut down, and take a woe is me attitude, and feel bad for yourself, and sit there and do nothing. But having a community of people that I can look to for inspiration, pick me up if I’m feeling down, or whatever, just this sense of community, has really kinda helped me kinda put my arms around my new life, and kind of embrace it.”

Beyond the sense of inclusion and belonging noted by the quotes above, participants voiced a sense of authenticity that they could only find once joining the AASA. The bonds formed through amputee soccer cross beyond the shared LD/LL and into the realm of considering each other as family.

Jose (39 y.o. male,15+ years’ experience with the AASA, transtibial) describes this sense of belonging:

“I feel more free to be authentic and to be myself than in other communities. I’m not part of that many other communities, I mean, there’s professional communities where you’re not always fully authentic. And so, this just offers a community where you can be authentic as an amputee.”

Max (30 y.o. male, 6-10 years’ experience with the AASA, transtibial) shared his sentiments about amputee soccer being more than just a team:

“I think what community means to me is your tribe. And your tribe is the people that enter your life and they're your chosen family. They're not your blood, but they're people that you understand you both have a purpose and an ability to serve each other for. Beyond that, it’s—specifically to amputee soccer—it’s a community that we all can relate to. It’s a level of being able to relate to someone that’s not anything I can feel with my nondisabled friends, with my nondisabled family. It’s only something that I can feel with them. And I think the uniqueness of it, and the nuance of it, to be able to have that, is something special. And there’s—it’s invaluable.”

Laura (37 y.o. female, 2-5 years’ experience with the AASA, knee disarticulation) shared her sentiments about support groups and how soccer is different:

“Oh, yeah. I tried to do the whole amputee groups, where you sit and you talk about your feelings, and you talk about your amputation a thousand times. To me, that made me more depressed because now I’m just sitting in my situation, versus the team sports, now I’m in it, and I’m in it to win it. “

Overall, the theme of inclusivity, belongingness, and authenticity is characterized by a sense of community that participating in amputee soccer brings. The selected participant quotes highlight the impact the sport has on their ability to feel like they are part of a community and have shared experiences with like individuals found by participating in amputee soccer. The community is unique and allows the participants to feel like their authentic self.

#### Theme 2: benefits body and mind

The physical nature of the sport is very demanding, and as a result participants are able to push their bodies to grow stronger and more capable. However, this function and capability expands beyond the playing field and can carry over into everyday life.

A quote from Sally (44 y.o. female, <1 year of experience with the AASA, hip disarticulation) shed light on the carryover from the field to life:

“My husband is more comfortable with me independently traveling, or going, and doing something that’s physically challenging with the kids; I’ll take them for hikes. And so, [amputee soccer] has not just improved my life, but in some ways, it’s improved his life, and my child’s life.”

Rose (38 y.o. female, <1 year of experience with the AASA, hip disarticulation) describes how the physical demands of the sport bolsters autonomy:

“To be a part of a community that yells at you to go faster, and yells at you to stand back up when you fall down, that want you to fall down because that means you’re pushing yourself. To have you do agility drills, and when you drop your crutches, they don’t all swarm to you because they [would] drop their crutches, too. They almost automatically know that you can take care of yourself, that you are independent. Especially if you’re wanting to participate in a sport, that you have certain abilities, and you’re okay. It’s almost depressing to leave the community of soccer players and go home.”

Beyond the physical, our participants reaped numerous mental health benefits from being part of amputee soccer. Max (30 y.o. male, 6-10 years’ experience with the AASA, transtibial) shared how his participation in amputee soccer has helped him overcome barriers in his life:

“This sport and the community that it’s opened doors for me to, has allowed me to heal from a mental health side. I think it’s been such an amazing journey to be able to walk through this life experience and have positive role models and mentors, people who look like me. To be able to guide and shape my—and help me not only cope with my disability, but also to thrive with it.”

Josh (29 y.o. male, 15+ years’ experience with the AASA, hip disarticulation) contributed how participation in amputee soccer helped him develop control and ownership of his life:

“The mentality of becoming 1% better every day and I think also developing an internal locus of control where you believe that you are the actor that is causing outcomes rather than outcomes are being played upon you as an object. So, I think just taking ownership over what you can control and allowing the things that you're unable to control to not impact your decision-making and your focus, and determination, and schedule and training. All those things I think you learn from playing soccer.”

In summary, this theme illustrates how participation in amputee soccer benefits both body and mind. Participants report how they are better able to engage in their lives, develop autonomy, and enhance resilience through their involvement.

#### Theme 3: call to action: advocating for others

Analyzing the transcripts in this study revealed an interesting theme that focused on the need to advocate for the advancement of the sport. Members of the amputee soccer community felt strongly that they played a role in the furtherment of the sport, and as such, took this responsibility seriously. The aspirations for the sport transcended beyond the competition itself, focusing on both accessibility and awareness for future players and individuals with limb loss/difference.

Sally (44 y.o. female, <1 year of experience with the AASA, hip disarticulation) shared her long-term goal for making the sport accessible:

“Our goal is that we will be in the Paralympics 2032,” My goal is not the World Cup, my goal is not being a better athlete. My goal is to do everything that I can to make this a Paralympic sport so that women and girls like me who have limb difference into the future have a big, huge, beautiful goal that they can focus on to rehabilitate themselves, to make them happy.”

Gary (60 y.o. male, 15+ years’ experience with the AASA, transtibial) illustrated his hope for the future generation of participants:

“Once I’m gone, and that’s what I want, is I want the kids coming up—5, 6 years old that may be born with a limb difference or trauma—that they have something to look forward to. That their world is not over, it’s only begun, and they can accomplish a lot.”

For Josh (29 y.o. male, 15+ years’ experience with the AASA, hip disarticulation), he shared how the sport can transform lives and wants to see this continue:

“It [amputee soccer] gives me purpose, gives me a life’s mission. And it’s not just me. There are plenty of people around the country and around the world who are seeing this as their life’s goal and there are plenty of people who are seeing this as a second chance and a way back to feeling whole again. So, I think that there is something more than just soccer going on in this sport. I think it provides genuine representation and humanity for a community that is oftentimes marginalized, particularly in other parts of the world. I think that the ability for soccer to be a vehicle for social change on a number of topics including disability is significant.”

Commenting on how growing the sport validates the players, Max (30 y.o. male, 6-10 years’ experience with the AASA, transtibial) shared:

“We had the opportunity to play at Gillette Stadium, which was our first full exhibition match in a professional stadium with about 500 fans. And just a huge step and win for amputee soccer in this country. Because for the first time, we weren't—no shade intended- we weren't a sideshow. We were the main show.”

Luis (31 y.o. male, <1 year of experience with the AASA, ankle disarticulation) articulately paints a picture of the slow, but progressive, nature of building the sport.

“I think that each one of us, we are building and leaving a little grain of sand and impacting the sport globally and nationally a little bit.”

In total, the participants in this study found a call to action when it came to advocating for the growth of the sport. The participants wanted other individuals like themselves to have access and be included in the sport so that they can benefit physically, mentally, and socially

## Discussion

The purpose of this study was to explore how participating in amputee soccer as a member of the AASA impacted biopsychosocial function. Three themes were generated from the coded interviews: community, physical and mental benefits, and advocacy. Our participants rated their quality of life as “good” to “very good” and “agreed” to “strongly agreed” that playing amputee soccer has improved their sense of community and belonging, confidence, energy levels, overall life satisfaction, and sense of purpose.

Our participant pool reflected a younger (average age <40 years old), primary male, highly functioning group of individuals. The primary reasons for limb loss were wholly consistent with either trauma or disease processes, primarily at the transfemoral and transtibial levels, with most individuals being involved in amputee soccer for less than 5 years. This population is likely representative of competitive amputee soccer in general as the rigors of the sport typically trend towards a particular demographic base (16). There were no significant differences between participants based on their demographic characteristics and their survey responses, as the distribution of variance with homogeneous.

Community in the context of this study can be thought of as a sense of belonging with others like themselves and is a key component of the social domain in the biopsychosocial model. Individuals with disabilities can frequently feel they do not belong in society for a number of reasons, including barriers to access, body image issues, or occupational restrictions (17, 18). This is well documented in the LD/LL population, with numerous studies reporting a sense of isolation due to their condition, which in turn can contribute to psychosocial disorders like depression and anxiety (19). While individuals with LD/LL may find a sense of community through participation in support groups (20), it may not always be enough to satisfy a sense of normality. In our study, we found that participants in amputee soccer had an increased sense of belonging that was uniquely different from what they may have experienced by participating in support groups alone. A study by Havlin et all found that by participating in amputee sports, an enhanced sense of belonging and normality was experienced by the individuals with LD/LL (21). Mueller et al. found that participation in adaptive sports compliments other interventions, building community and a sense of belonging. Another aspect of amputee soccer participation that contributes to the sense of community is that players have similar physical attributes, specifically, the ability to use crutches if they have unilateral LD/LL, or in the case of an individual with LD/LL of the upper extremity, the ability to tend the goal using the uninvolved extremity. Players typically need to have sufficient fitness to navigate the pitch, therefore, many of the participants in the sport are both demographically and physically more similar than different. These findings reinforce our results that participation in amputee soccer offers benefits from other (non-adaptive sport) interventions, including the building a supportive community that provides benefits not typically experienced elsewhere.

The benefits one experiences by participating in amputee soccer went beyond just the more obvious physical benefits that are associated with playing a sport. Studies have shown that participating in amputee soccer will yield improvements in strength and balance, decrease pain, and improve overall physical function (22, 23). Our study corroborates these other findings, supporting that through amputee soccer an individual with LD/LL can improve their function and abilities. However, our study also found benefit to the psychological domain through participation. Some of our participants felt that through participation in amputee soccer that they were able to transcend beyond their disability into becoming more self-efficacious and that it helped them reframe life with LD/LL. Other studies looking at the benefits of adaptive sports have found similar findings, with an increase in self-efficacy, quality of life, confidence, and psychological resilience being a result of their participation (24–27). This increase in confidence and self-efficacy has been shown to be transferrable to other aspects of daily life, showing the benefit of participating in adaptive sports beyond the field (25, 28). Since many of the hardships experienced while playing the sport are similar to that in everyday life with a disability, having a safe and supportive environment to grow and learn is invaluable to an individual with LD/LL. This finding adds to the body of literature that adaptive sport interventions should be a consideration as first line treatments for particular populations, in addition to traditional support groups.

The theme of advocacy was an interesting finding from this study. Participants in our study felt a strong sense of ownership over the sport and wanted to make it available to as many individuals with LD/LL as possible. This was particularly due to the benefits they gleaned from participating in the sport and the urge to encourage others to join so that they could benefit from participation. Advocacy happens primarily through the showcase of athleticism. Known as the “Paralympic paradox”, advocacy efforts are geared both towards able-bodied and disabled individuals simultaneously (29). Purdue & Howe describe this paradox as the tension created by athletes wanting to both be recognized as elite athletes, yet, also be valued for their role as a disabled athlete. This is supported by the remarks of one of our participants in the study who referred to the playing of the sport as the “main show” and not the “side show,” indicating that there is value in being seen as both different and capable. However, participation in the sport also fosters alternative ways to advocate. One of the primary ways our participants advocated for the sport is through mentoring others (30). Mentoring was a strategy that helped individuals with disabilities overcome their own doubts about their capabilities, as well as provide a sense of satisfaction knowing that they are helping someone else like themselves. In this way, much like one our participants stated, they are leaving the sport better than when they joined. This finding is unique in that it shows development of one’s sense of ownership of the sport, something that is important when living with a chronic condition (31).

As with any qualitative study, there are limitations intrinsic to the methodology. While great care was taken to be objective in the interview, coding, and theme formation, the threat of researcher bias remains. We attempted to mitigate the threat by having the individual responsible for the interviewing not participate in the coding. We also used the consensus method for the coding, theme formation, and selection of representative quotations. The sample was restrictive by the very nature of the inclusion criteria and the purpose of the study, therefore, not all findings from this study may be transferable to other populations.

In conclusion, participants in this study reported that playing amputee soccer brought a sense of community, physical and mental benefits, and a strong call to advocate for the sport so that others can benefit in the future. Participation in the sport brought biopsychosocial benefits beyond what could be experienced in a support group alone.

## Data Availability

The datasets presented in this article are not readily available because data is from a very specific population and includes potentially identifying data intrinsic to the interview transcripts. Requests to access the datasets should be directed to daniel.lee.8@stonybrook.edu.
