# Supplementary material for: The biopsychosocial benefits of amputee soccer: perspectives from members of the American Amputee Soccer Association
Source: Front Sports Act Living. 2026 Mar 25;8:1791418. doi: 10.3389/fspor.2026.1791418 (PMC13057551; doi:10.3389/fspor.2026.1791418)
Supplement: Supplementary file 1 [file Supplementaryfile1.pdf]

Appendix A: Demographic Form

Age

____

Gender

____

Work status

- Employed Full Time
- Employed Part Time
- Not Employed

Occupation(s) -- Please include all current employed occupations

____

Level of limb loss

- Ankle disarticulation (At the ankle)
- Knee disarticulation (At the knee)
- Transfemoral (Above the knee)
- Transtibial (Below the knee)
- Hip or Pelvic disarticulation (At the hip)
- Wrist disarticulation (At the wrist)
- Transradial (Below the elbow)
- Elbow disarticulation (At the elbow)
- Transhumeral (Above the elbow)
- Shoulder disarticulation (At the shoulder)
- Forequarter (Above the shoulder)

Cause of limb loss

- Diabetes
- Birth
- Infection
- Trauma
- Cancer
- Vascular

Time since loss of limb

- 0-1 year
- 2-5 years
- 6-10 years

- 11-15 years
- 16-20 years
- 20+ years

Hours of prosthesis use (daily average)

- 0-2 hrs/day
- 3-5 hrs/day
- 6-8 hrs/day
- 9-11 hrs/day
- 11-13 hrs/day
- 14+ hrs/day

What sports do you currently play?

What sports have you played in the past?

What is the highest level of competition you have ever competed at?

- National Team
- Regional Team
- Recreational
- Unsure

How did you find out about amputee soccer?

- Friend/family
- Internet
- Prosthetist
- Therapist
- Medical doctor
- Print article

How long have you played amputee soccer?

- 0-1 year
- 2-5 years
- 6-10 years
- 11-15 years
- 15+ years

Appendix B: Researcher Questions

Please indicate the extent to which you agree with the statements. Likert scale ranging from 1 (Strongly disagree) to 5 (Strongly agree):

Quality of life/inclusivity

1. Amp soccer has improved my overall life satisfaction.
2. Amp soccer has improved my engagement and interest in leisure activities.
3. Amp soccer has improved my sense of community.
4. Amp soccer improved my support network.
5. Amp soccer has improved my sense of belonging.

Self- efficacy

6. Amp soccer has improved my overall self-confidence.
7. Amp soccer has improved my overall perception of yourself.
8. Amp soccer has improved my sense of purpose.

Health

9. Amp soccer has improved my overall physical fitness.
10. Amp soccer has improved my physical activity.
11. Amp soccer has improved my energy levels.

Appendix C: WHOQOL-BREF

You would circle number 1 if you did not get any of the support that you needed from others in the last two weeks. Please read each question, assess your feelings, and circle the number on the scale for each question that gives the best answer for you.

THE WHOQOL-BREF

		Very poor	Poor	Neither poor nor good	Good	Very good
1	How would you rate your quality of life?	1	2	3	4	5

		Very dissatisfied	Dissatisfied	Neither satisfied nor dissatisfied	Satisfied	Very satisfied
2	How satisfied are you with your health?	1	2	3	4	5

The following questions ask about **how much** you have experienced certain things in the last two weeks.

		Not at all	A little	A moderate amount	Very much	An extreme amount
3	To what extent do you feel that (physical) pain prevents you from doing what you need to do?	1	2	3	4	5
4	How much do you need any medical treatment to function in your daily life?	1	2	3	4	5
5	How much do you enjoy life?	1	2	3	4	5
6	To what extent do you feel your life to be meaningful?	1	2	3	4	5

		Not at all	A little	A moderate amount	Very much	Extremely
7	How well are you able to concentrate?	1	2	3	4	5
8	How safe do you feel in your daily life?	1	2	3	4	5
9	How healthy is your physical environment?	1	2	3	4	5

The following questions ask about **how completely** you experience or were able to do certain things in the last two weeks.

		Not at all	A little	Moderately	Mostly	Completely
10	Do you have enough energy for everyday life?	1	2	3	4	5
11	Are you able to accept your bodily appearance?	1	2	3	4	5
12	Have you enough money to meet your	1	2	3	4	5

	needs?					
13	How available to you is the information that you need in your day-to-day life?	1	2	3	4	5
14	To what extent do you have the opportunity for leisure activities?	1	2	3	4	5

		Very poor	Poor	Neither poor nor good	Good	Very good
15	How well are you able to get around?	1	2	3	4	5

The following questions ask you to say how **good or satisfied** you have felt about various aspects of your life over the last two weeks.

		Very dissatisfied	Dissatisfied	Neither satisfied nor dissatisfied	Satisfied	Very satisfied
16	How satisfied are you with your sleep?	1	2	3	4	5
17	How satisfied are you with your ability to perform your daily living activities?	1	2	3	4	5
18	How satisfied are you with your capacity for work?	1	2	3	4	5
19	How satisfied are you with yourself?	1	2	3	4	5
20	How satisfied are you with your personal relationships?	1	2	3	4	5
21	How satisfied are you with your sex life?	1	2	3	4	5
22	How satisfied are you with the support you get from your friends?	1	2	3	4	5
23	How satisfied are you with the conditions of your living place?	1	2	3	4	5
24	How satisfied are you with your access to health services?	1	2	3	4	5
25	How satisfied are you with your transport?	1	2	3	4	5

The following question refers to **how often** you have felt or experienced certain things in the last two weeks.

		Never	Seldom	Quite often	Very often	Always
26	How often do you have negative feelings such as blue mood, despair, anxiety, depression?	1	2	3	4	5

Appendix D: Interview Questions

1. How does playing amputee soccer make you feel?
2. How has amputee soccer impacted your quality of life?
3. How does being part of a team make you feel?
4. How does playing soccer in front of a crowd make you feel?
5. What has amputee soccer taught you?
6. How has amputee soccer impacted your view on disability and other people with disability?
7. How has being part of the amputee soccer community affected you?
8. How does amputee soccer compare to other sports you may have played?
9. Do you feel like you play a role in the growth of amputee soccer around the US and world?
10. What is one of your most memorable moments involving amputee soccer?
11. What barriers exist to participating in amputee soccer?
12. What facilitators exist to participate in amputee soccer?
13. Is there anything else you would like to add about amputee soccer and how it has impacted your life?
